# Targeted lung denervation in sheep: durability of denervation and long-term histologic effects on bronchial wall and peribronchial structures

**DOI:** 10.1186/s12931-020-01383-3

**Published:** 2020-05-18

**Authors:** Martin L. Mayse, Holly S. Norman, Alexander D. Peterson, Kristina T. Rouw, Philip J. Johnson

**Affiliations:** Nuvaira, Inc, Suite 105 3750 Annapolis Lane North, Minneapolis, MN 55447 USA

## Abstract

**Background:**

Targeted lung denervation (TLD), a novel bronchoscopic procedure which attenuates pulmonary nerve input to the lung to reduce the clinical consequences of neural hyperactivity, may be an important emerging treatment for COPD. While procedural safety and impact on clinical outcomes have recently been reported, the mechanism of action has not been reported. We explored the long-term pathologic and histopathologic effects in a sheep model of ablation of bronchial branches of the vagus nerve using a novel dual-cooled radiofrequency ablation catheter.

**Methods:**

Nineteen sheep underwent circumferential ablation of both main bronchi with simultaneous balloon surface cooling using a targeted lung denervation system (Nuvaira, Inc., USA). Animals were followed over an extended time course (30, 365, and 640 days post procedure). At each time point, lung denervation (axonal staining in bronchial nerves), and effect on peribronchial structures near the treatment site (histopathology of bronchial epithelium, bronchial cartilage, smooth muscle, alveolar parenchyma, and esophagus) were quantified. One way analysis of variance (ANOVA) was performed to reveal differences between group means on normal data. Non-parametric analysis using Kruskal-Wallis Test was employed on non-normal data sets.

**Results:**

No adverse clinical effects were observed in any sheep. Nerve axon staining distal to the ablation site was decreased by 60% at 30 days after TLD and efferent axon staining was decreased by >70% at 365 and 640 days. All treated airways exhibited 100% epithelial integrity. Effect on peribronchial structures was strictly limited to lung tissue immediately adjacent to the ablation site. Tissue structure 1 cm proximal and distal to the treatment area remained normal, and the pulmonary veins, pulmonary arteries, and esophagus were unaffected.

**Conclusions:**

The denervation of efferent axons induced by TLD therapy is durable and likely a contributing mechanism through which targeted lung denervation impacts clinical outcomes. Further, long term lung denervation did not alter the anatomy of the bronchioles or lung, as evaluated from both a gross and histologic perspective.

## Background

Chronic obstructive pulmonary disease (COPD) is a significant health burden, which is anticipated to grow in prevalence [1, 2]. COPD is characterized by persistent airflow obstruction due to (1) increased bronchoconstriction, (2) increased mucous secretion, and (3) hyperresponsive airways, which largely results from long-term exposure to particulates or may be related to chronic virus or infections [3]. These pathophysiologic characteristics result in part from increased parasympathetic input to the lungs, which causes a heightened pathological response in the airways. Specifically, the parasympathetic nervous system modulates airway resistance by regulating airway smooth muscle tone and mucus secretion through stimulation of airway submucosal glands [4–8]. Pulmonary branches of the vagus nerve travel along the adventitial layers of the mainstem bronchi to the lungs [9].

Long-acting pharmacologic blockade of cholinergic nervous input (parasympathetic input) has become a mainstay of treatment for COPD, resulting in marked improvements in dyspnea, frequency of exacerbations, and lung function by relaxing chronically constricted airways [10–12]. Unfortunately deposition of these inhaled anticholinergic agents to the most diseased areas of the lungs is not possible due to decreased ventilation in those locations [13]. Airway caliber and alveolar ventilation are decreased in exacerbating COPD patients [14, 15]. This change likely confounds inhaled therapy distribution and may contribute to the continued incidence of exacerbations reported in up to 70% of patients on optimal inhaled triple therapy [16]. Surgical denervation of pulmonary vagal inputs has resulted in marked decreases in airway resistance [6, 17–22]; however, the high morbidity and mortality associated with surgical denervation due to difficulty in approaching the pulmonary branches of the vagus nerve [23] has limited the utility of this approach. A less invasive and safer way to reliably ablate the pulmonary inputs of the vagus would therefore represent a promising novel therapeutic strategy in COPD.

Targeted Lung Denervation (TLD) seeks to ablate the bronchial vagal nerve fibers to denervate the lung, via a minimally invasive procedure which has been described in detail elsewhere [20]. Denervation decreases basal smooth muscle tone in the airways, may also reduce inflammation and mucous secretion [24], and blunts airway hyperresponsiveness [25–27] . In particular, airway hyperresponsiveness, has been linked to pathologic modulation of the parasympathetic nervous system in response to local stimuli (limited to a single region of the lung), such as viral or bacterial infection [28]. This pathologic modulation facilitates a whole lung response that resembles the characteristics of an exacerbation of obstructive lung diseases. Thus, durable denervation of the parasympathetic input to the lung has the potential to impact exacerbations.

In early clinical studies TLD has been shown to be safe and effective [29–32]. Most recently, a double-blinded, randomized, sham controlled, trial of 82 COPD patients demonstrated a lower rate of respiratory related adverse events in the TLD treatment arm compared to control arm (32% vs. 71%, *p* = 0.0008) and demonstrated a lower risk of COPD exacerbation requiring hospitalization in the TLD group [29]. It is hypothesized that the reduction in respiratory events and severe COPD exacerbations is directly related to durable denervation; however, limited basic science evidence of the durability of denervation following TLD is available.

The current study aims to determine the durability of lung denervation following TLD, to investigate any long term adverse impacts to the anatomical structure of the lung and to provide insight into the mechanism through which TLD might impact COPD exacerbations. Prior studies have demonstrated that TLD denervates the lung and is associated acutely with decreased nerve input to the lung. Disruption of efferent input was confirmed by a decrease in airway resistance, and loss of sensory input was confirmed by loss of the Herring-Brewer reflex. Acute preservation of the bronchial mucosa at TLD treatment sites, and prevention of excessive collateral damage to other critical peribronchial structures (e.g., esophagus, major blood vessels, heart, and lung) was reported out to 30 days in these earlier studies [20]. The current manuscript details the pathologic and histopathologic effects of TLD in a sheep model, with animals followed out to 640 days after the procedure.

## Methods

### Study design

Nineteen sheep (37 airways) underwent the TLD procedure in both main bronchi with simultaneous balloon surface cooling using a lung denervation system (Nuvaira, Inc., USA), as previously described in detail [20]. All studies were conducted under the guidance of an Institutional Animal Care and Use committee. Treatment of the animals was in accordance with study facility’s standard operating procedures, which adhere to the USDA Animal Welfare Act (9 CFR, Parts 1, 2 and 3) and The Guide for Care and Use of Laboratory Animals (ILAR publication, 1996, National Academy Press).

Sheep were selected without regard to sex, with weights between 40 and 80 kg. Animals were followed over an extended time course: 30 (*n* = 6), 365 (*n* = 5), and 640 (*n* = 8) days post procedure, with 4 control sheep (7 airways) exposed to no treatment for specified analyses. The design of this study was chosen to allow the proximal location, which is unaffected by the procedure to be used as a control for each animal, excluding ChAT analysis, which allowed for the lowest number of animals sacrificed. The TLD treatment effect has been previously shown to be distal to the ablation zone, with proximal locations outside the zone of tissue ablation representative of healthy control tissue [20]. At each time point, gross evaluation of tissue, surface protection at the treatment site (histopathology of bronchial epithelium), effect on bronchial and peribronchial structures near the treatment site (histopathology of bronchial cartilage, vasculature, smooth muscle, alveolar parenchyma, and esophagus) and lung denervation (axonal staining in bronchial nerves) were quantified at the treatment site, distal to the treatment site, and proximal to the treatment site with the proximal site serving as a control for each animal.

### Targeted lung denervation procedure

The Targeted Lung Denervation (TLD) procedure performed has been described in detail previously [20]. Briefly, procedures were performed under general anesthesia using IV and/or inhaled anesthetics, following a 24-h fast. Animals were intubated with a large endotracheal tube (10 mm internal diameter). The radiofrequency (RF) ablation system was positioned in the mainstem bronchus and activations were performed at eight different rotational positions within the airway. Once activations were completed, the ablation catheter was directed into the contralateral bronchus, which was then treated in similar fashion. Ablation was performed for 120 s at each location using 20 W (W) at all locations except at the three posterior octants (dorsal, dorsomedial, and dorsolateral positions) in the left mainstem bronchus where power was reduced to 15 W to avoid potential injury to the esophagus due to the vagal nerve trunks which follow that structure to innervate the gastrointestinal system.

### Gross evaluation and histologic tissue preparation

Animals were euthanized following bronchoscopy at the end of the designated follow-up period. The chest cavity was opened and the heart, pericardium, aorta, esophagus, mediastinum and lungs were evaluated grossly. Any observed gross lesions were trimmed for processing and evaluation. If no gross lesions were noted in the mediastinum, aorta, or heart, these tissues were not evaluated histologically. The lungs, bronchi, thoracic trachea, and esophagus were then excised and instilled with 10% neutral buffered formalin at an inflation pressure of 20 to 25 cm water to allow fixation near their normal expanded state. Following 2–4 days fixation the right and left lung fields and associated main bronchi were separated at the midline. The medial wall of each bronchus was incised in the horizontal plane to allow opening of the airway and gross visualization of the mucosal surface of each bronchus. Each bronchus was then trimmed into 9–12 sequential cross sections approximately 5 mm in thickness.

The right and left main vagal trunks that run along the esophagus were identified distal to the tracheal bifurcation and tagged with suture to assist in identification during trimming. Sections of right and left vagal trunks and adjacent esophageal wall distal to the treatment sites were trimmed. Multiple adjacent cross-sections of the esophagus were trimmed in the region where it passes over the left mainstem bronchus, to evaluate for collateral damage to the esophagus at this site.

All trimmed tissues were then processed through graded alcohols, cleared in xylene, embedded in paraffin, sectioned at 5 μm and stained with hematoxylin & eosin (H&E) for light microscopic evaluation. The sections were then analyzed by a blinded evaluator as described below to quantify the histologic effects of the TLD procedure.

### Histologic assessment of bronchial and peribronchial structures

**Fig. 1:**
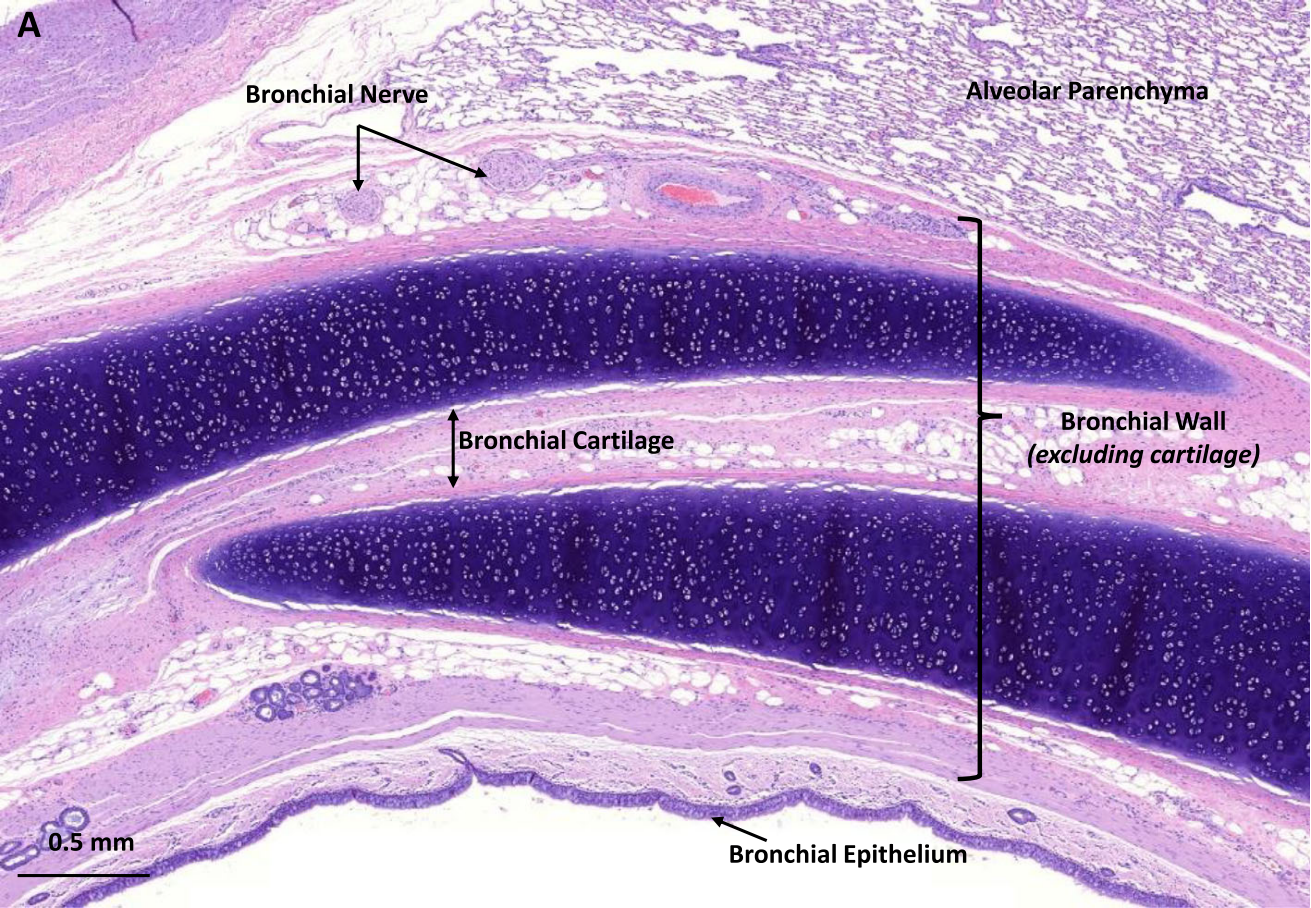
Representative histochemical section from outside of the TLD treatment zone in a sheep 30 days post-procedure. Grouping of bronchial structures for semi-quantitative analysis. The tissue that makes up and immediately surrounds the airway was divided into four separate structures for analysis. The bronchial epithelium was scored as a single tissue. The bronchial lamina propria, smooth muscle, submucosa, and adventitial layer between airway and alveolar tissue made of the tissue scored as the bronchial wall. The bronchial cartilage was scored independent of the bronchial wall. Bronchial nerves were scored using immunohistochemical analysis and a nerve located in the adventitia that immediately surrounds the airway is shown (scale bar is 0.5 mm)

Histologic changes at the treatment site were evaluated on two sections per airway (proximal to the treatment site and at the treatment site), unless otherwise specified, using a semi-quantitative grading scheme in the airway wall and surrounding tissues (Fig. 1). The severity (0: No histologic change, 1: Minimal, 2: Mild, 3: Moderate, 4: Severe) and type (categorized as Necrosis, Acute Inflammation, Chronic Inflammation, and Fibrosis) of tissue effects were evaluated within the bronchial epithelium, bronchial wall (defined as the lamina propria, smooth muscle, submucosa, and adventitial layer between airway), bronchial cartilage, surrounding alveolar parenchyma, surrounding blood vessels (the pulmonary arteries and veins), and esophagus. Each treated airway was assigned a pathology score based on this grading scheme. In addition to these four types of tissue effects, epithelium was also assessed for hyperplasia and metaplasia, alveolar parenchyma for airway ectasia, and blood vessels for neo-intimal proliferation and thrombosis.

The percentage of the airway circumference at the treatment site at the time of airway harvest (one or more sections) that was lined by viable epithelium was determined by visual estimation. This assessment was defined as the epithelial integrity of the airway following treatment.

### Immunohistochemical assessment of lung denervation

Immunohistochemical staining was performed on select airway cross sections. A commercial monoclonal antibody cocktail called Pan Neuronal Marker (pNM) (1:200 dilution, Millipore Corp, Billerica, Mass, USA) was used to identify all axonal subtypes (i.e. efferent and sensory axons) running along the airway in cross sections at each treatment site as well as proximal and distal to the treatment site. A monoclonal antibody against choline acetyltransferase (ChAT;1:100 dilution, Vector Labs, Burlingame, CA, USA) was used to identify parasympathetic efferent axons that run along the airway in cross sections distal to the treatment site. Negative control staining was performed on at least one section of airway by omitting the primary antibody for both types of immunohistochemical staining.

A semiquantitative assessment was developed to evaluate the extent of axonal staining with the Pan Neuronal Marker in nerve fascicles surrounding the airways prior to and after the TLD procedure in three sections per airway: proximal to the treatment site, at the treatment site, and distal to the treatment site. Axon staining intensity within individual nerve fascicles of 50 μm or greater in diameter was scored from 0 to 10 using the following semiquantitative scale: 0 = no staining of any axon fibers; 1 = staining of <= 10% of the fascicle; 2 = staining of > 10 and < = 20% of the fascicle; …; 9 = staining of > 80 and < = 90% of the fascicle; 10 = staining of > 90% of the fascicle. The score of stained tissue sections proximal to the site of treatment were used as controls of normal staining and were compared to the score of stained tissue sections at and distal to the site of TLD treatment (Fig. 6). Proximal tissue sections can be used as controls due to the normal behavior of axons following nerve injury which results in the axons distal to the site of injury undergoing the process of Wallerian degeneration and those proximal to the site of injury remain structurally intact [33].

Due to the small percentage of efferent axons present in bronchial nerve fibers [34], the precision of the semi-quantitative axonal assessment described above was insufficient for analysis of ChAT stained efferent axons (Fig. 7). Scoring was instead performed via image analysis of fascicles using Image J software (2018/1.51 W) to quantify the percent of the fascicle area stained on one section per airway distal to the treatment location for TLD treated animals, and at a similar location from control animals. Airway cross sections distal to the treatment site were divided into quadrants with up to three representative fascicles quantified per quadrant. The average of up to 12 sampled fascicle scores in a cross section was used as the overall score for the airway. Cross sections from the same approximate anatomical location in untreated airways were used as a control for normal ChAT expression because the expression of ChAT following nerve injury is dynamic [35–37].

### Statistical analysis

All data are reported as mean ± standard error (SEM) except where otherwise specified. One way analysis of variance (ANOVA) was performed to reveal differences between group means on normal data sets (*p* < 0.05 indicates significance). Non-parametric analysis using Kruskal-Wallis Test was employed on non-normal data sets. If differences were observed, Tukey’s test was used to determine pairwise differences between group means.

## Results

Overall, there were no clinically evident complications from the TLD procedure and all sheep survived until their scheduled necroscopy. Blood cell counts, chemistries, and arterial blood gases showed no significant abnormalities when compared to known values for sheep in captivity (data not shown).

*Gross histologic effect of TLD.*

No significant gross lesions were identifiable in the lungs of any of the animals. Gross histologic effects at 30 days have been previously reported [20]. By 365 days post TLD, mild fibrotic replacement of parenchyma (only 1-2 mm thick) adjacent to the treatment site identified at 30 days [20] appeared to resolve and the treatment sites were difficult to identify grossly due to minimal visible tissue effects. At 365 days, one airway of the ten presented with a small tissue thickening of the lumen approximately 5 cm distal from the carina. By 640 days post TLD, sixteen of sixteen treated bronchi presented no significant changes in the airway lumen architecture at the treatment site and the treatment site was grossly indistinguishable from normal bronchi. Further, no obvious gross changes were observed in airway architecture due to injury to cartilage, and no evidence for airway stenosis or malacia was observed in any airways (data not shown).

There were no serious effects in the major pulmonary arteries or veins reported at any time point, although mild changes, including localized mild mural fibrosis and localized mild neointimal proliferation were noted out to 365 days and absent after 640 days. There were no gross treatment-associated effects identified in either the right and left main vagal branches distal to the treatment site at any time point.

No serious effects were identified in the esophagus at any time point following treatment, indicating no attenuation of the vagal nerve trunks responsible for innervation to the gastrointestinal system. In two animals at 30 days, mild to minor focal fibrosis affecting a very limited area of the outer tunica muscularis was identified, suggesting focal damage to the muscularis in the outer layer of the esophageal wall during treatment. No esophageal effects were observed after the 30-day time point out to 640 days. Gross inspections of the heart, pericardium, aorta, and mediastinum revealed no treatment related effects at any time point post TLD (data not shown).

### General histologic effects of TLD on bronchial wall

**Fig. 2:**
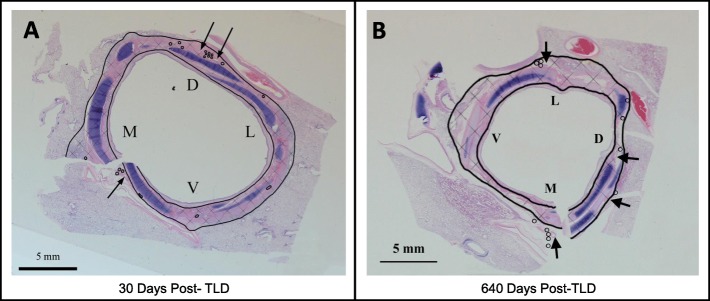
Representative histochemical cross-sections at the treatment location at (**a**) 30 days and (**b**) 640 days post TLD. H&E staining was used to delineate tissue structures in the bronchi. An inner and outer black line delineate the zone of ablation evident by a dense fibrotic scarring in the tissue and highlighted by the cross hatched region in the image. The region between the drawn inner black line and the airway surface is a zone of epithelial protection provided by the cooled balloon and electrode of the catheter. The arrows indicate mucosal glands and the circles indicate nerve fascicles. The letters V, M L, and D indicate the ventral, medial, dorsal, and lateral aspect of the airway, respectively

**Fig. 3:**
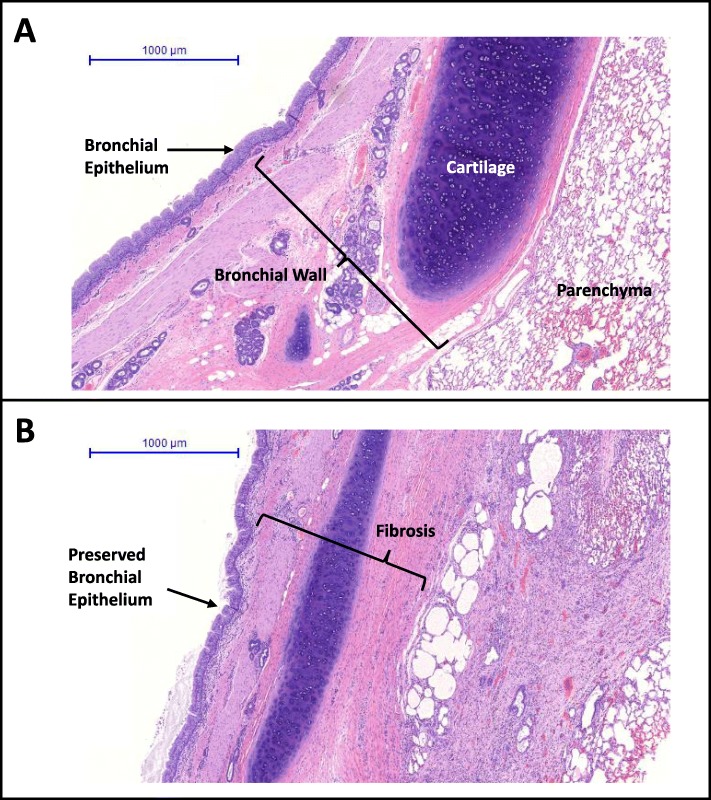
Representative high magnification histochemical sections at the (**a**) proximal and (**b**) at the treatment site. Annotations indicate preserved bronchial epithelium and fibrosis

Compared to proximal tissue samples which represent an intra-animal control, the TLD treatment effect consisted of a band of remodeled tissue in the outer layers of the bronchial wall and the surrounding adventitia (Fig. 2 demonstrated this for 30 and 640 days post-TLD, cross hatched region). This band of remodeled tissue is largely circumferential but is limited along the length of the airway to the region of contact with the electrode. It consisted of well-organized fibroplasia extending outward from the submucosa into the surrounding adventitia and often entrapped and occasionally obliterated normal structures including the targeted nerves, submucosal glands, small blood vessels and bronchial cartilage (Fig. 3). Moderate to severe fibrosis was consistently observed through the adventitial layers of the bronchi (where branches of the vagus nerve are located). The depth of fibrosis from the airway wall was stable over time and did not change out to 640 days (Fig. 2b), confirming durability of the TLD treatment effect.

The band of fibrosis and remodeled tissue was typically separated from the lumen of the airway by a protected layer which often included normal bronchial epithelium, lamina propria, and some smooth muscle tissue (Fig. 2, inner line).

### Histologic effects on bronchial and peribronchial structures

#### Bronchial epithelium

**Fig. 4:**
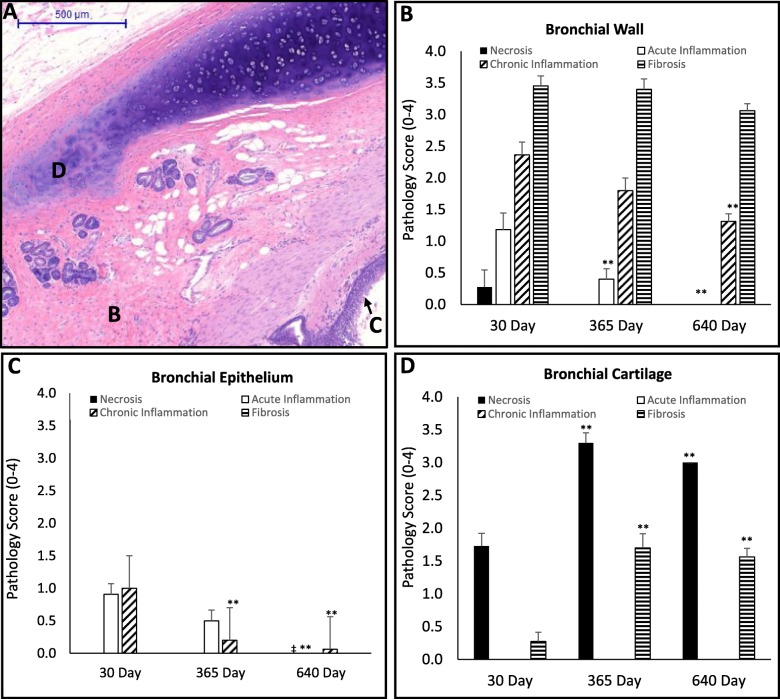
(**a**) Representative histologic cross section of cartilage necrosis, bronchial wall fibrosis, and preserved bronchial epithelium. (**b**-**d**) Histopathological analysis at 30 days (*n* = 6 animals, 11 airways), 365 days (*n* = 5 animals, 10 airways), and 640 (*n* = 8 animals, 16 airways) of the bronchial anatomy for necrosis (black bars), acute inflammation (open bars), chronic inflammation (diagonal stripe bars) and fibrosis (horizontal stripe bars). Data are presented as mean ± SEM. *p < 0.05 vs 30 day time point, **p < 0.001 vs 30 day timepoint, † *p* < 0.05 365 day vs 640 day time point, ‡ *p* < 0.001 365 day and 640 day time point. (**b**) Semi-quantitative analysis of bronchial wall. The adventitia surrounding the bronchi is the target of TLD therapy because of the presence of bronchial nerves. The semi-quantitative analysis of the bronchial wall at the treatment site, which include the adventitia, demonstrates consistently moderate levels fibrosis over the 640 day time course. Acute inflammation decreased (*p* < 0.001) at 365 day vs. 30 day and remained low for the time course. Chronic inflammation decreased (*p* < 0.001) at 640 days vs. 30 day timepoint. (**c**) Semi-quantitative analysis of bronchial epithelium. The bronchial epithelium at the treatment site demonstrates only minimal treatment related tissue changes that largely resolve over the 640 day time course. Acute inflammation decreases significantly (*p* < 0.001) at the 640 days versus the 30 day timepoint. Chronic inflammation decreases significantly (*p* < 0.001) at both 365 and 640 days versus the 30 day timepoint. (**d**) Semi-quantitative analysis of bronchial cartilage. The cartilage in sheep are located directly below the TLD electrode and are subject to energy levels that are not intended to occur in the human lung. In this way, the sheep serves as a sensitive model that amplifies TLD related tissue effects in the cartilage. Necrosis and fibrosis were elevated (*p* < 0.001) at the 365 day time point and remained stable out to 640 days

The bronchial epithelium is protected from radiofrequency (RF) energy delivered from the Nuvaira catheter by coolant circulating through the catheter electrode and balloon. This protection is evident during histologic analysis by a layer of unaffected or minimally affected tissue extending from the surface of the airway into the wall (Fig. 2, inner line; Fig. 3b). This minimal effect is also evident in the histopathology scores for the bronchial epithelium (Fig. 4). Necrosis and fibrosis were absent throughout the 640-day time course. Minimal inflammation (both acute and chronic) were observed at 30 days and decreased over time. Proximal tissue was absent of procedural effects (Table 1). Gross evaluation of bronchial epithelium immediately proximal and distal to the treatment site was absent of all tissue effects (data not shown).

**Table 1:** Histochemical semi-quantitative evaluation of proximal location to treatment site. Hematoxylin-eosin staining was used to evaluate the structural changes. All sections were evaluated on semi-quantitative 1–4 point scale. Values are mean ± SEM

Tissue	Observation	30 Day (*n* = 6 animals, 11 airways)	365 Day (*n* = 5 animals, 10 airways)	640 Day (*n* = 8 animals, 16 airways)
Bronchial Epithelium	Necrosis	0.0 ± 0.0	0.0 ± 0.0	0.0 ± 0.0
	Acute Inflammation	0.5 ± 0.2	0.2 ± 0.1	0.0 ± 0.0
	Chronic Inflammation	0.5 ± 0.2	0.2 ± 0.1	0.1 ± 0.1
	Fibrosis	0.0 ± 0.0	0.0 ± 0.0	0.0 ± 0.0
Bronchial Wall	Necrosis	0.0 ± 0.0	0.0 ± 0.0	0.0 ± 0.0
	Acute Inflammation	0.5 ± 0.2	0.1 ± 0.1	0.0 ± 0.0
	Chronic Inflammation	1.7 ± 0.2	1.2 ± 0.1	1.1 ± 0.1
	Fibrosis	0.5 ± 0.2	0.4 ± 0.1	0.3 ± 0.1
Bronchial Cartilage	Necrosis	0.0 ± 0.0	0.0 ± 0.0	0.0 ± 0.0
	Acute Inflammation	0.0 ± 0.0	0.0 ± 0.0	0.0 ± 0.0
	Chronic Inflammation	0.0 ± 0.0	0.0 ± 0.0	0.0 ± 0.0
	Fibrosis	0.0 ± 0.0	0.0 ± 0.0	0.0 ± 0.0
Alveolar Parenchyma	Necrosis	0.0 ± 0.0	0.0 ± 0.0	0.0 ± 0.0
	Acute Inflammation	0.0 ± 0.0	0.0 ± 0.0	0.0 ± 0.0
	Chronic Inflammation	1.0 ± 0.0	1.0 ± 0.0	1.0 ± 0.0
	Fibrosis	0.0 ± 0.0	0.0 ± 0.0	0.0 ± 0.0
	Hyperplasia	0.0 ± 0.0	0.0 ± 0.0	0.0 ± 0.0
	Metaplasia	0.0 ± 0.0	0.0 ± 0.0	0.0 ± 0.0
	Airway Ectasia	0.0 ± 0.0	0.0 ± 0.0	0.0 ± 0.0

#### Bronchial wall

Durable denervation of the lung requires disruption of efferent axons and formation of fibrosis within bronchial nerve branches located in adventitial layers surrounding the bronchial wall to prevent reinnervation. Targeting of the outer layer of the bronchial wall with formation of fibrosis is clearly achieved and is demonstrated by the extent of fibrosis observed in these tissues (Fig. 4b). Moderate fibrosis was consistently reported by 30 days and remained stable 640 days out from TLD therapy. Chronic inflammation in the bronchial wall was mild on average at 30 days following TLD therapy and decreased at 365 days, achieving significance by day 640 (*p* < 0.001). Acute inflammation was minimal at 30 days and absent by 640 days. Tissue necrosis was largely absent across all time points. Proximal tissue was absent of procedural effects (Table 1). Gross evaluation of the bronchial wall distal to the treatment site demonstrated the normal minimal level of chronic inflammation (data not shown).

#### Bronchial cartilage

The bronchial cartilage is located within the outer layers of the bronchial wall but was scored as a separate tissue type because of its distinctive anatomy in the sheep and its unique response to TLD therapy. The cartilage in sheep bronchi consists of overlapping plates that are distributed along the length of the treated bronchi and, as such, some cartilage is always situated directly below the TLD electrode. This sheep-specific anatomy subjects the cartilage to the highest intensity of the delivered energy and provides a very sensitive model of cartilage tissue effects. The sheep cartilage structure is unlike that reported in the main bronchi of humans, where the airway cartilage forms discrete rings between which the Nuvaira electrode is designed to sit. This design will minimize the effect of TLD on cartilage rings in humans.

In the sheep, cartilage necrosis was observed by 30 days, which became moderate in intensity at 365 days and remained stable from that time out to 640 days post TLD therapy (Fig. 4d). Additionally, fibrosis was noted at mild levels at 365 days and remained stable over the remainder of the time course. Chronic and acute inflammation was absent over the time course observed.

Cartilage proximal and distal to the treatment site displayed no pathological effects of treatment (Table 1, Figs. 1 and 3a). Given the ring structure of cartilage in the human airway, it is expected that the cartilage rings proximal and distal to the actual treatment site will be subjected to minimal effect from TLD therapy.

#### Alveolar parenchyma

**Fig. 5:**
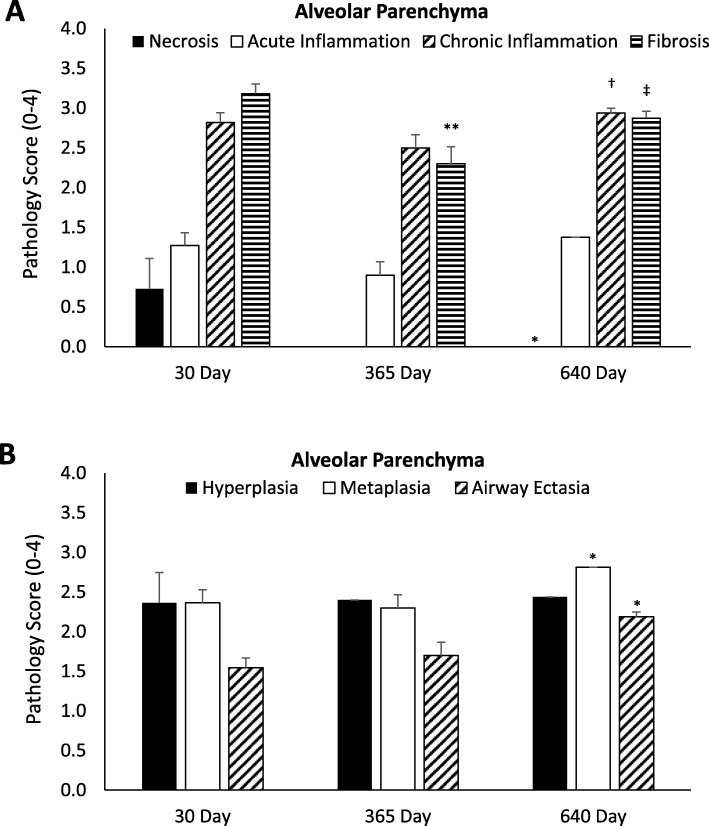
Histopathological analysis at 30 days (*n* = 6 animals, 11 airways), 365 days (*n* = 5 animals, 10 airways), and 640 (*n* = 8 animals, 16 airways) of the alveolar parenchyma. Data are presented as mean ± SEM. *p < 0.05 vs 30 day time point, **p < 0.001 vs 30 day timepoint, † *p* < 0.05 365 vs. 640 day time point, ‡ *p* < 0.001 365 vs 640 day time point. **a** Semi-quantitative analysis of Alveolar parenchyma for necrosis (black bars), acute inflammation (open bars), chronic inflammation (diagonal stripe bars) and fibrosis (horizontal stripe bars). A small volume of alveolar parenchyma demonstrates tissue related effects of TLD therapy. **b** Semi-quantitative analysis of Alveolar parenchyma for hyperplasia (black bars), metaplasia (open bars) and ectasia (diagonal stripe bars). A small volume of alveolar parenchyma demonstrates tissue related effects of TLD therapy

In some locations surrounding the TLD treatment site, alveolar parenchyma is situated within close proximity to the outer edge of the bronchial wall. TLD therapy often affected alveolar tissue surrounding the treatment site to a depth of less than 2.2 mm. Mild to moderate fibrosis, airway ectasia, hyperplasia, metaplasia and chronic inflammation were all observed in the small portion of alveolar tissues surrounding the treatment site. These findings are evident at the very first observation post treatment (30 days), with chronic inflammation and fibrosis varying slightly between the 365 and 640 day timepoints. Minimal acute inflammation is also apparent by 30 days and remains stable over the entire time course. Necrosis is largely absent following the 30 day timepoint (Fig. 5 a-b).

The scoring of the parenchymal changes was limited to the areas of affected tissue and disregarded the large amount of unaffected alveolar parenchyma adjacent to the treatment sites. The average depth of parenchyma affected by TLD therapy was less than 2.2 mm. Considering this depth, the average length of alveolar parenchyma affected, and the radius of the treated airways, the total volume of alveolar parenchyma affected by TLD was calculated to be approximately maximum 8 × 10-4 L. With a normal lung volume in the sheep of approximately 3.5 L, on average less than 0.05% of the lung tissue was affected by TLD therapy.

The alveolar tissue immediately proximal and distal to the treatment site was essentially normal (Table 1). There was minimal chronic inflammation over the entire 640-day time course that is a common finding in sheep lung and is not a result of TLD therapy. All other findings were absent.

### Immunohistochemical assessment of lung denervation

#### Axonal analysis with Pan neuronal marker

**Fig. 6:**
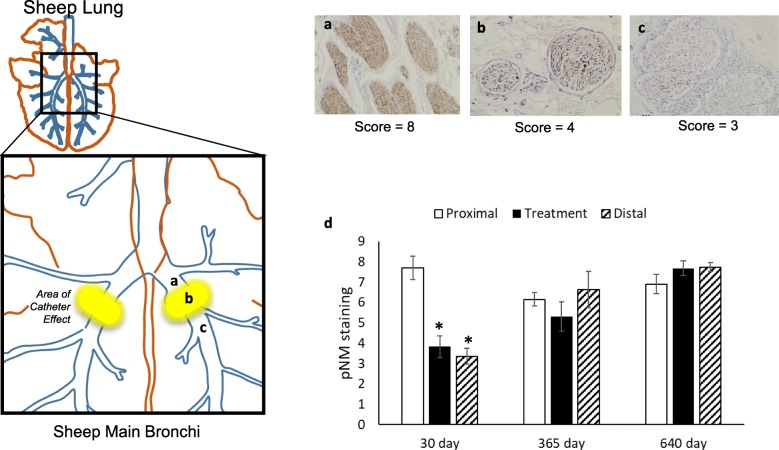
Pan-Neuronal Marker (pNM): (**a)** proximal, (**b**) activation site and (**c**) distal to the site of TLD therapy. (**d**) Semi-quantitative analysis of pNM staining in TLD treated nerves at 30 days (*n* = 6 animals, 11 airways), 365 days (*n* = 5 animals, 10 airways), and 640 days (*n* = 8 animals, 16 airways) at proximal to treatment site (open bars), treatment site (black bars), and distal to treatment site (diagonal stripe bars). At 30 days post TLD, a significant decrease in bronchial nerve expression of pNM demonstrates significant disruption of bronchial axons following TLD. Data are presented as mean ± SEM, *p < 0.001 vs proximal control

Immunohistochemical analysis using a Pan Neuronal Marker (pNM) was used to quantify the effect of TLD therapy on efferent and sensory axons located within the targeted bronchial nerves. The marker in part identifies neurofilament expression in both efferent and sensory axons. Neurofilament is a cytoskeletal protein that provides the structural support for axons and is unique to neuronal cells [38]. Following injury, the expression of neurofilament is maintained in the proximal portion of the injured axon and is lost at the site of injury and in the distal component of the axon. At 30 days following TLD therapy, there was a significant decrease in pNM expression at the site of treatment and distal to the treatment site when compared to proximal expression (*p* < 0.05, Fig. 6). Treatment site and distal pNM expression returned to proximal expression values by the 365-day time point.

The analysis of pNM expression was also performed in the main trunks of the vagus nerve running along the esophagus distal to the treatment location. Power was dropped (15 W) in treatment locations near the main trunks of the vagus nerve as a safety factor to prevent inadvertent disruption. The main trunks were evaluated in 19 sheep at 30 days (*n* = 6), 365 days (*n* = 5), and 640 days (*n* = 8) after treatment. All of examined vagus nerve trunks demonstrated normal pNM staining (19 of 19) showing that the main trunks of the vagus nerve are unaffected by TLD treatment.

#### Efferent axon analysis with ChAT

**Fig. 7:**
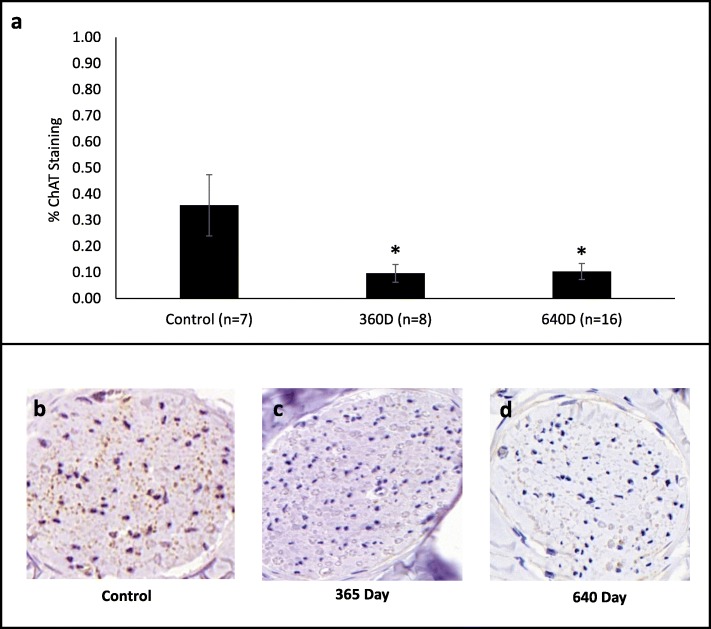
Semi-quantitative analysis of ChAT staining in TLD treated nerves over a 640 day time course. (**a**) Consistent decrease in ChAT staining in the distal airways at 365 (*n* = 8 airways) and 640 (*n* = 16 airways) days compared to untreated distal airways (*n* = 7 airways) demonstrates durable disruption of efferent axons that innervate the lung. Data are presented as mean ± SEM, *p < 0.05 vs proximal control. Representative ChAT staining in a control airway (**b**) and in treated airways at 365 days (**c**) and 640 days (**d**)

Choline acetyltransferase (ChAT) is a protein in the nervous system which in nerve tissue is found exclusively within acetylcholine producing cells and is highly concentrated in efferent neurons. Immunohistochemical staining for this protein and morphometric assessment allows for tracking disruption and or regeneration of efferent axons within nerve fascicles surrounding the airway following TLD therapy. Airway cross sections distal to the treatment site were used for animals evaluated at 365 and 640 days. Cross sections from the same approximate anatomical location in untreated airways were used as a control for the ChAT analysis. ChAT expression in the control airways distal to the treatment site was sparse with 0.36 ± 0.12% (mean ± SEM) of fascicle area containing positively stained axons. ChAT staining presented significantly decreased expression of 0.10 ± 0.03% in the 365 day group (Fig. 7, *p* = 0.031) demonstrating disruption of efferent axons at 1 year. This 73% reduction in ChAT expression after TLD therapy remained stable at 640 days suggesting long term absence of regeneration.

## Discussion

The present study demonstrated effective denervation of parasympathetic efferents contained within the pulmonary branches of the vagus with no long-term detrimental effects to the animal, to airway morphology, or other peribronchial structures. TLD therapy would ideally disrupt regeneration of these efferent axons by producing fibrosis in and around the nerve fascicles and axons. Fibrosis, or scarring, of the tissue surrounding and separating the proximal and distal stumps of an injured nerve has been shown to impede nerve regeneration. Following nerve injury, a repair cascade is initiated to contain damage, organize the distal stump, and prepare for axonal regeneration. This sequence of events includes an upregulation of TGF-beta, which among other mechanisms, modulates fibroblast proliferation, stimulating overproduction of extracellular matrix proteins, including the major component of scar tissue, collagen. While a normal part of the repair process, if excessive scar is formed due to excessive perineural fibrosis, axonal regeneration is disrupted [39, 40]. TLD was previously demonstrated to produce excessive scar formation between the proximal and distal stump of an injured nerve [20]. Evidence of efferent denervation was shown up to 640 days with the downregulation of ChAT (Fig. 7), which was likely enabled by the ring of fibroplasia that surrounds the airway as a result of TLD. The depth and intensity of fibrosis appears to be stable by 30 days and does not change significantly for up to 640 days after TLD (Figs. 2, 3, and 4). Thus, it appears that TLD produces an adequate lesion depth to reliably and durably ablate pulmonary branches of the vagus nerve, without negatively impacting the integrity of the bronchial wall.

### Effect on bronchial and peribronchial structures

To assess the durable effects of efferent neuron denervation on bronchial anatomy, the bronchial and peribronchial structures were evaluated for impact on bronchial anatomy. Previously, the acute effect of the TLD procedure was published, highlighting safe and effective vagal denervation, as well as an acute downregulation of the pan-neurofilament [20]. Further, recent data was published highlighting the safe and effective profile of the Nuvaira TLD system [30]. Results presented here confirm long-term viability of the bronchial and peribronchial structures following TLD treatment and durable denervation out to 640 days.

### Durability of efferent neuron denervation

It is known that nerves are involved in the pathophysiology of obstructive lung disease such as COPD and asthma, as bronchoconstriction, mucus production, and airway hyperresponsiveness are CNS modulated [28, 41]. Further, local viral infections are associated with changes in the control of the neurotransmitter, acetylcholine, release in airway smooth muscle, which translates into global obstructive lung disease exacerbations [28]. Therefore, denervation of the vagal nerve would be expected to impact these characteristics, positively impacting clinical outcomes. In the current study, assessment of choline acetyltransferase (ChAT) was performed to evaluate efferent axon disruption to the lung. Choline acetyltransferase is a protein found in nerve exclusively within efferent neurons and is not present in sensory neurons; therefore, assessment of ChAT is a reliable surrogate for loss of efferent function [42–46]. Acetylcholine-releasing axons of efferent neurons, the primary target of TLD therapy, are relatively few in number in bronchial nerves but significantly influence smooth muscle contraction and bronchoconstriction in COPD [34]. ChAT staining distal to the treatment site was decreased by 73% by 365 days and remained stable at 640 days suggesting that TLD therapy provides durable disruption of the efferent component of bronchial nerve fibers (Fig. 7).

In contrast to ChAT, neurofilament is a cytoskeletal protein which provides structural support for both afferent and efferent axons. Following injury, the expression of neurofilament is maintained in the proximal portion of the injured axon and is lost in the distal component of the axon which is cleared during the process of Wallerian degeneration. The transient ~ 75% decrease in pan-neurofilament staining distal to the treatment site observed at 30 days confirms the TLD nerve disruption demonstrated with ChAT staining. Recovery of pan-neurofilament to pre-treatment levels at 365 days, combined with the sustained loss of efferent staining, suggests regeneration of sensory axons to a point immediately distal to the site of treatment.

Previous studies assessing recovery from vagus nerve injury suggest that efferent axons are generally more sensitive to barriers in axonal regeneration than sensory axons [47]. While the exact mechanisms that lead to these differences have yet to be identified, factors that may affect the regenerative differences include efferent and sensory axon competition in which afferent axons outnumber efferent axons, anatomical differences in neuronal soma location with efferent bodies located in the brainstem and afferent cell bodies located in the nodose ganglia in the neck, and metabolic differences in the regenerative cascade. Following subdiaphragmatic transection of the abdominal vagus nerve in rats, sensory reinnervation of the gut was apparent at 45 weeks while efferent reinnervation failed [48]. In the present study, dense fibrosis in the targeted region of the airway wall is demonstrated in parallel with decreased ChAT staining in nerve fascicles distal to TLD treatment out to 640 days. Conclusions about the function of regenerating sensory fibers reported in the current study cannot be made; however, it is generally known that anatomic recovery does not necessarily translate to physiologic recovery. It is unknown whether or not these fibers successfully reinnervated the whole lung or were only successful in traversing the short gap distal to the treatment site that was evaluated histologically. Efferent and sensory nerve function is required for an adequate parasympathetic response, future studies will have to evaluate the functionality of sensory regeneration following TLD in the presence of decreased efferent innervation.

## Conclusion

TLD has previously been shown to be safe and recent data indicates a positive clinical impact through decreased COPD exacerbations [29–32]; however, the mechanism of action requires further elucidation. This study demonstrates that TLD can create durable denervation of vagal efferent fibers while protecting bronchial epithelial surface and minimizing impact to other peribronchial structures. Efferent neuron staining showed absence of regeneration out to 640 days. Overactive parasympathetic nerve input to the lungs is implicated in the pathophysiology of COPD [7] and exacerbations of COPD [28, 49]. The current report provides evidence that TLD durably disrupts parasympathetic nerve input to the lung of treated sheep and may provide insight into the mechanism through which TLD reduces exacerbations in treated patients with obstructive lung disease.

## Data Availability

The datasets used and/or analyzed during the current study are available from the corresponding author on reasonable request.

## Abbreviations

ANOVA: Analysis of variance
ChAT: Choline acetyltransferase
CNS: Central nervous system
COPD: Chronic obstructive lung disease
H&E: Hematoxylin & eosin
pNM: Pan-neuronal marker
SEM: Standard error of the mean
TLD: Targeted lung denervation
USDA: United States department of agriculture
W: Watts
